# Skull Base Squamous Cell Carcinoma Presenting as Collet-Sicard Syndrome: A Rare Cause of Multicranial Nerve Dysfunction

**DOI:** 10.7759/cureus.78237

**Published:** 2025-01-30

**Authors:** Nirosha Metta, Ananya Lade, Jayashankar CA, Ganaraja V Harikrishna, Shravan S Sanjee

**Affiliations:** 1 Neurology, Vydehi Institute of Medical Sciences and Research Centre, Bangalore, IND; 2 Internal Medicine, Vydehi Institute of Medical Sciences and Research Centre, Bangalore, IND; 3 Neurology, National Institute of Mental Health and Neuro Sciences, Bangalore, IND; 4 General Medicine, Vydehi Institute of Medical Sciences and Research Centre, Bangalore, IND

**Keywords:** base of the skull, carotid sheath, collet-sicard syndrome, lower cranial nerve palsy, squamous cell carcinoma (scc)

## Abstract

The lower cranial nerves (CNs) - IX, X, XI, and XII - are affected by lesions of the skull base that impact the hypoglossal canal and jugular foramen in Collet-Sicard Syndrome (CSS), a rare disorder. Loss of posterior tongue taste sensation (IX nerve), paralysis of the vocal cords and difficulty swallowing (resulting from the X nerve), weakness in the sternocleidomastoid and trapezius muscles (due to the XI nerve), and atrophy and weakness of the tongue muscles (caused by the XII nerve) are all signs of this condition.

The purpose of this report is to describe a case of squamous cell carcinoma (SCC), presenting features of multiple CN palsies.

We report a 61-year-old gentleman presenting with hoarseness of voice, whispering speech, gradually progressive difficulty in swallowing both solids and liquids, associated with nasal regurgitation of food, difficulty in formation of food bolus, and weakness of the right shoulder for six months. Neurological examination showed a left-deviated uvula, absent gag reflex on the right side, atrophied and weak right sternocleidomastoid muscle, weaker right-sided shrug, drooping of the right shoulder, and atrophy of the right side of the tongue, which was deviated towards the right. Contrast-enhanced computed tomography (CECT) of the head and neck showed heterogeneously enhancing lesions in the region of the right vallecula and base of the tongue, extending medially to cross the midline and involving the opposite vallecula, a few enlarged, heterogeneously enhancing, necrotic right cervical lymph nodes, and an abutting right submandibular gland completely encasing the carotid sheath (CS) and narrowing its contents. A biopsy of the lesion revealed well-differentiated SCC. Management involved surgical resection of the lesion and concurrent chemoradiation. The patient had symptomatic relief from her symptoms and was able to swallow liquids without any difficulty at a two-month follow-up.

We emphasize that skull base lesions like SCC should be considered in the differential diagnoses of patients presenting with CNs IX-XII palsies. This also highlights the importance of multidisciplinary care in patients with multiple CN palsies.

## Introduction

Collet-Sicard Syndrome (CSS) is a rare syndrome involving unilateral palsy of the lower cranial nerves (CNs) IX, X, XI, and XII, due to a lesion affecting the jugular foramen and hypoglossal canal. Frederic Collet initially reported it in 1915 as glossolaryngo-scapulopharyngeal hemiplegia, due to damage of the lower four CNs [1].

Although it was first described a century ago, recently many causes have been described with CSS. The most frequent causes of the lesions are dissections of the carotid arteries and basilar skull fractures (due to damage to structures near the jugular foramen and hypoglossal canal, especially CNs) [2]. Examples of iatrogenic causes include cerebral angiography, internal jugular vein catheterization, cerebral vessel clamping, and glomus jugulare tumors, which can sometimes damage nearby CNs [3,4]. Neoplastic causes include parotid tumors, hypoglossal nerve schwannomas, tumors of the base of the skull, and metastases. Other non-neoplastic etiologies include infectious and inflammatory causes, such as osteomyelitis of the skull base, Trousseau syndrome, and chronic otitis media, which can also damage nearby structures due to the infective and inflammatory processes, especially lower CNs [5]. Sometimes, this form of multiple CN palsies is seen even with polyarteritis nodosa, varicella-zoster, and Lyme disease [6,7]. This highlights the list of various CSS etiologies and the importance of comprehensive evaluation.

## Case presentation

A 61-year-old male, a chronic smoker for 40 years, presented with complaints of whispering speech, increased nasal twang (which increased on bending the head forward and resolved on lying on the back), gradually progressive difficulty in swallowing both solids and liquids, associated with nasal regurgitation of food, choking on his own saliva while talking, difficulty in the formation of food bolus, and weakness of the right shoulder for six months. The symptoms had slowly worsened for two months with no diurnal variations or fatigability. There is no history of trauma, fracture, decreased hearing or ringing sensation, headache, tremors, palpitations, recent catheterization, or any significant past or family history.

**Figure 1:**
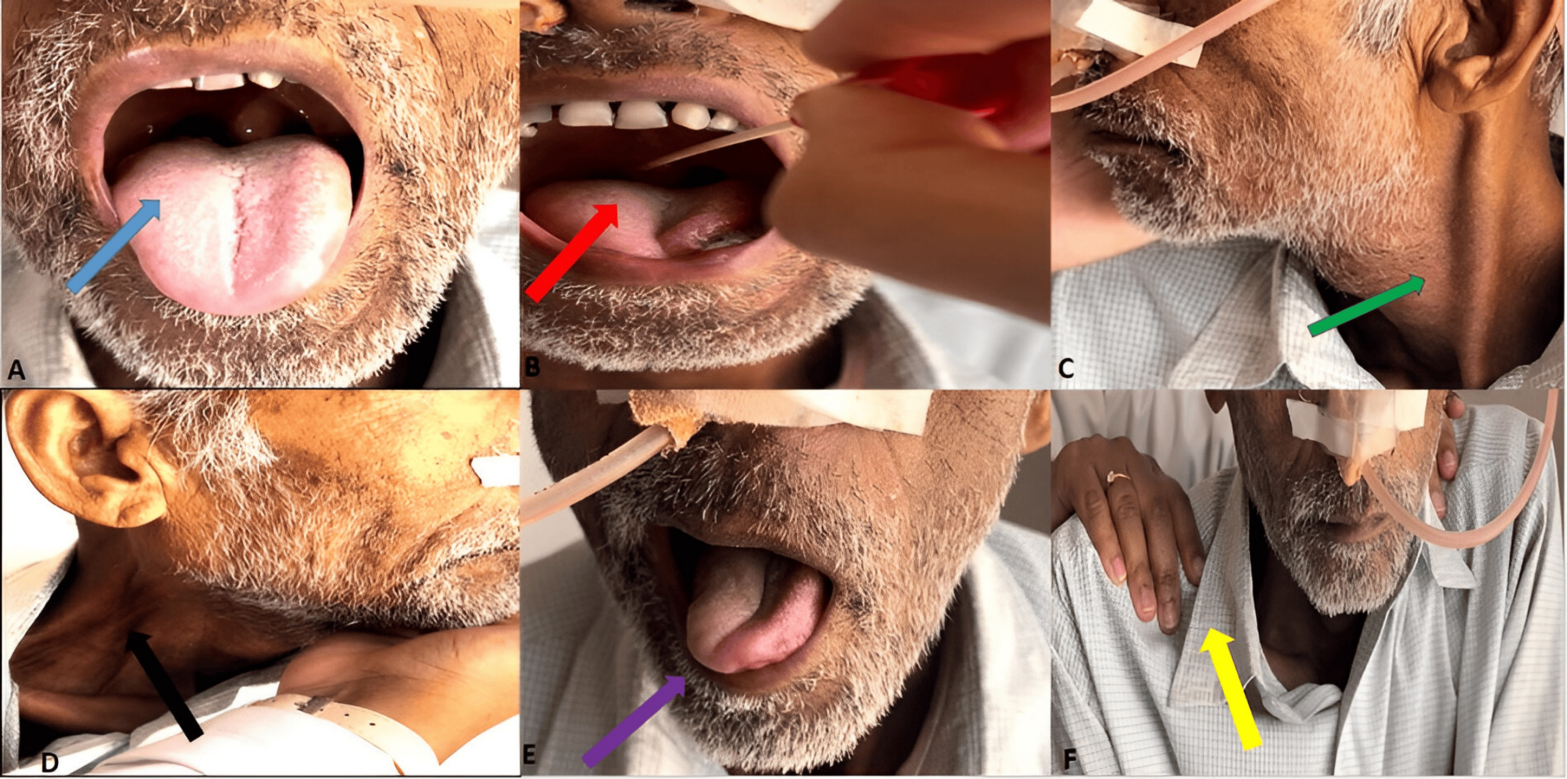
Patient showing features of (A) left-sided deviated uvula (blue arrow), (B) absent right gag reflex (red arrow), (C) prominent left sternocleidomastoid muscle on resistance (green arrow), (D) absent right prominent sternocleidomastoid muscle with resistance (black arrow), (E) deviated tongue to the right side (purple arrow), and (F) drooping of the right shoulder (yellow arrow).

On examination, his vital signs were stable. Neurological examination revealed deviation of the uvula to the left side, absent gag reflex on the right side, prominent left sternocleidomastoid muscle on resistance, and absent right prominent sternocleidomastoid muscle with resistance, atrophy of the right side of the tongue, which was deviated towards the right, and drooping of the right shoulder with the prominent right clavicle (Figure 1). The rest of the examination was normal.

**Figure 2:**
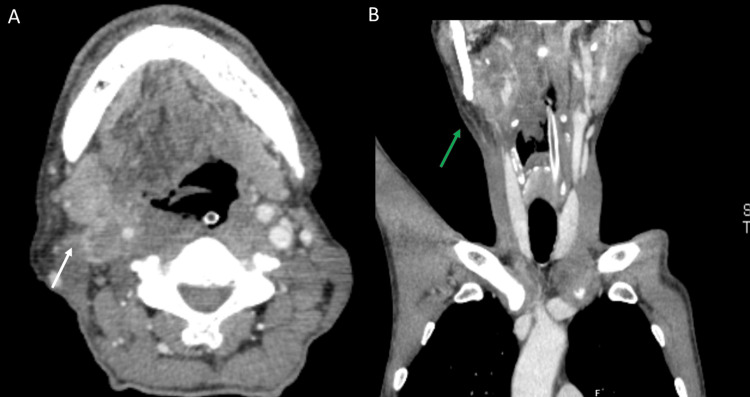
CT scan of the head and neck showing (A) lymph nodes at the right submandibular gland (white arrow), and (B) a heterogeneously enhancing lesion in the region of the right vallecula and base of the tongue (green arrow). CT: Computed Tomography

The patient's biochemical parameters, including hemogram, liver, thyroid, kidney function tests, and serum electrolytes, were within normal limits. A computed tomography (CT) scan of the head and neck with contrast showed enhancing necrotic lymph nodes measuring 33 x 21 x 43 mm, seen abutting the right submandibular gland and completely encasing the carotid sheath (CS), narrowing its contents, and a heterogeneously enhancing lesion in the region of the right vallecula and base of the tongue, extending medially to cross the midline and involve the opposite vallecula (Figure 2). The surgical oncologist and otorhinolaryngology opinions were sought for further management and tissue diagnosis.

**Figure 3:**
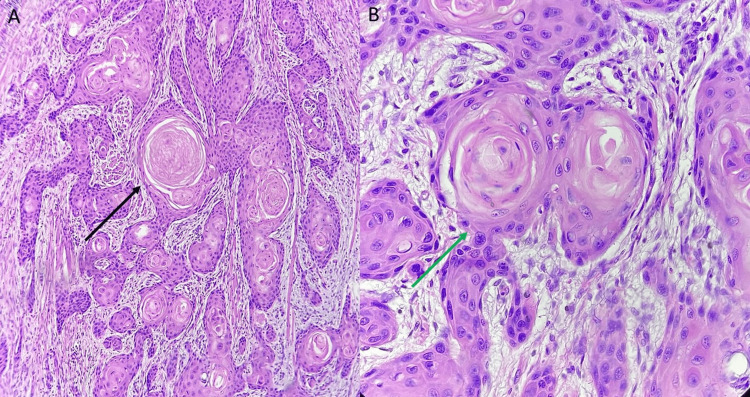
Biopsy from the base of the tongue showing (A) irregular tissue bits lined by stratified squamous epithelium (black arrow), and (B) tumor cells seen infiltrating into the underlying sub-epithelium in clusters (green arrow).

A biopsy from the base of the tongue showed irregular tissue bits lined by stratified squamous epithelium, showing hyperplasia and focal moderate dysplasia. Tumor cells were seen infiltrating into the underlying sub-epithelium in clusters. These were large polygonal cells with abundant eosinophilic cytoplasm and vesicular nuclei, with prominent nucleoli. Dyskeratosis and a few keratin pearls were also noted (Figure 3).

The patient was started on concurrent chemoradiotherapy with weekly cisplatin 40 mg/m², and radiotherapy of 70 Gy/33#, followed by surgical resection. He responded well to the treatment and started taking a liquid diet at two months of follow-up.

## Discussion

CSS, described by Collet and Sicard, is an uncommon condition characterized by simultaneous unilateral paralysis of CNs IX, X, XI, and XII [1]. At times, it manifests incompletely, resulting in conditions known as jugular foramen syndrome (JFS), due to overlapping clinical features in both conditions. The CSS diagnosis is based on clinical history, physical examination, and neuroimaging. In our case, IX, X, XI, and XII CN palsies are caused by a base of skull lesion impinging on the CS and its contents.

The pathophysiology of CSS is closely linked to the pathway of the four lower CNs as they travel through the jugular foramen and alongside the major blood vessels in the neck, due to their close relation with the CS. The CS is an important anatomic structure in the head and neck, housing various crucial neurovascular elements, such as the carotid artery, jugular vein, vagus nerve, and sympathetic plexus. This fibrous connective tissue sheath extends from the arch of the aorta up to the base of the skull, surrounding the common carotid artery, internal carotid artery (ICA), internal jugular vein, CNs IX, X, XI, and XII, the sympathetic nerve plexus, and deep cervical lymph nodes.

Tumors related to CS are predominantly cervical metastases, particularly squamous cell carcinomas (SCCs). These tumors can metastasize to the deep internal and external parajugular lymph nodes, leading to lymph node enlargement, as seen in our case. Primary lesions in the CS can be recognized by their displacement of the parapharyngeal space anteriorly. The causes of CSS encompass invasive conditions at the skull's base, including primary or metastatic tumors, trauma, vascular problems, inflammation, and complications from medical treatments. A study indicated that tumors are the leading cause of CSS, followed by vascular and traumatic factors [2,3,8,9].

CS paragangliomas are carotid body tumors, glomus jugulare tumors surrounding Jacobson's nerve (CN IX) or Arnold's nerve (the auricular branch of CN X), and glomus vagale tumors along CN X, invading the jugular foramen and potentially extending in a superior fashion, displacing the ICA anteriorly. Additionally, nerve sheath tumors, such as neurofibromas, schwannomas, and neuroblastomas, can also affect the CS [8,10]. Likewise, carotid artery aneurysms or arteriovenous malformations (AVMs) affecting the CS can also result in this syndrome. Although the specific tumor or vascular abnormality may influence the symptoms, they all share one key characteristic: compression of the CNs within the CS. Identifying the lesion’s source is critical for selecting the correct treatment approach [11].

In our case, it was initially suspected to be multiple CN palsy. On evaluation, the pattern was suggestive of CSS, which, on tissue diagnosis, was confirmed to be due to SCC. This SCC of the skull base, with extensions and necrotic lymph nodes compressing the right CS and its contents, was responsible for causing CSS on the right side. Although the differential diagnosis of glomus jugulare tumors, schwannoma, and nasopharyngeal carcinoma was considered, based on imaging findings and the need for a definitive diagnosis, tissue diagnosis by surgery was pursued. SCC of the base of the tongue, especially when it extends to the skull base, has the potential to cause CSS [12].

As reported by Bakst et al., glossopharyngeal (IX), vagus (X), and hypoglossal (XII) nerve palsies are frequently seen in later stages of primary nasopharyngeal tumors that extend into the jugular foramen or hypoglossal canal, causing symptoms such as hoarseness, difficulty swallowing, and loss of taste [13]. However, the accessory nerve (XI), often implicated in CSS, is typically unaffected unless the tumor reaches the more lateral regions of the skull base [13]. In our case, all four CNs were involved. In advanced stages of head and neck squamous cell carcinomas (HNSCCs), the tumor can spread to surrounding tissues and invade neural structures at the skull base, resulting in symptoms typical of CSS.

When CSS is suspected, the clinician should opt for a comprehensive evaluation, including imaging studies to ensure an accurate diagnosis, and also consult a surgical oncologist for tissue diagnosis if a neoplastic etiology is suspected. CT scans with or without angiography and magnetic resonance imaging with or without contrast are two possible imaging modalities for tumoral, vascular, and traumatic reasons.

As per Kohli and Gandotra, metastasis to the skull base and the upper cervical lymph nodes (Level II) from bronchogenic carcinoma resulted in CSS, as demonstrated in our case, where skull base carcinoma led to cervical Level II lymph node metastases and CSS [14]. In contrast, Shetty et al. presented a case of CSS caused by lymph node metastasis from lung adenocarcinoma, with no involvement of the skull base, affecting cervical lymph nodes 5 and 7, which is different from our case [11].

The treatment of CSS focuses on addressing the underlying cause. Management should be personalized for each patient, and a multidisciplinary team should be involved when necessary. The rates of CN functional recovery are typically highest for vascular and infectious causes, followed by traumatic etiologies [9]. This can be due to two primary factors. One factor, due to inflammation from close contact, can cause CN palsy, which may improve over time. Other factors are due to the mass effects of the lesion, like an intravascular clot or skull base abscess, or may also result in CN palsy in these cases. Adequate treatment to alleviate the mass effect on CNs can result in gradual improvement of symptoms. On the other hand, surgically removing malignancies around the jugular foramen is technically challenging. The majority of patients undergo subtotal excision followed by radiotherapy, and their fragile condition makes them more susceptible to postoperative complications [9]. The general prognosis for skull base tumors with CSS is poor, as is the recovery of the CN. In this case, surgical resection was followed by concurrent chemoradiotherapy, along with steroid administration to reduce edema, relieve symptoms, and enhance quality of life, leading to favorable survival rates [9]. These results align with the findings from the study by Tiwari et al. on SCC of the base of the tongue with surgical excision and postoperative radiotherapy [12].

## Conclusions

This case highlights the importance of considering CSS in patients with CN palsies in the context of skull base lesions, particularly SCC of the base of the tongue with extensions and necrotic lymph nodes compressing the right CS. Early recognition of this disease plays a crucial role in planning appropriate treatment. Overall management includes surgical resection, chemoradiotherapy, and supportive care, which can provide symptomatic relief and improve the quality of life in patients with this rare and debilitating syndrome.
